# Direct Restriction of Virus Release and Incorporation of the Interferon-Induced Protein BST-2 into HIV-1 Particles

**DOI:** 10.1371/journal.ppat.1000701

**Published:** 2010-03-05

**Authors:** Kathleen Fitzpatrick, Mark Skasko, Thomas J. Deerinck, John Crum, Mark H. Ellisman, John Guatelli

**Affiliations:** 1 Department of Medicine, University of California San Diego, La Jolla, California, United States of America; 2 Department of Neurosciences, University of California San Diego, La Jolla, California, United States of America; 3 National Center for Microscopy and Imaging Research, University of California San Diego, La Jolla, California, United States of America; 4 San Diego Veterans Affairs Healthcare System, San Diego, California, United States of America; Northwestern University, United States of America

## Abstract

Investigation of the Vpu protein of HIV-1 recently uncovered a novel aspect of the mammalian innate response to enveloped viruses: retention of progeny virions on the surface of infected cells by the interferon-induced, transmembrane and GPI-anchored protein BST-2 (CD317; tetherin). BST-2 inhibits diverse families of enveloped viruses, but how it restricts viral release is unclear. Here, immuno-electron microscopic data indicate that BST-2 is positioned to directly retain nascent HIV virions on the plasma membrane of infected cells and is incorporated into virions. Virion-incorporation was confirmed by capture of infectivity using antibody to the ectodomain of BST-2. Consistent with a direct tethering mechanism, we confirmed that proteolysis releases restricted virions and further show that this removed the ectodomain of BST-2 from the cell surface. Unexpectedly, enzymatic cleavage of GPI anchors did not release restricted virions, weighing against models in which individual BST-2 molecules span the virion and host cell membranes. Although the exact molecular topology of restriction remains unsolved, we suggest that the incorporation of BST-2 into viral envelopes underlies its broad restrictive activity, whereas its relative exclusion from virions and sites of viral assembly by proteins such as HIV-1 Vpu may provide viral antagonism of restriction.

## Author Summary

The cellular protein BST-2 prevents newly formed particles of HIV-1 and other enveloped viruses from escaping the infected cell by an unclear mechanism. Here, we show that BST-2 is appropriately positioned to directly retain newly formed HIV-1 virions on the cell surface and is incorporated into infectious virions. We suggest that the incorporation of BST-2 into virions is a key aspect of the protein's broadly restrictive activity against enveloped viruses.

## Introduction

The innate defense against viruses includes specific host cell proteins with intrinsic abilities to restrict viral replication. In some cases these restriction factors have been linked to classic aspects of the innate immune response, such as the antiviral state induced by type I interferons. To replicate in this hostile environment, viruses encode specific antagonists of restriction factors [1]. Several of the so-called accessory proteins of primate immunodeficiency viruses have been recognized as such antagonists. For example, the HIV-1 accessory protein Vpu was long known to enhance the release of progeny virions from infected cells, potentially by antagonizing an intrinsic cellular restriction to virion-release [2],[3]. The study of this phenomenon led to the discovery of the antiviral activity of a protein with no previously known function, BST-2 (also known as HM1.24 and CD317), now referred to as a “tetherin” based on its ability to retain nascent virions on the surface of infected cells [4]–[6]. BST-2 is an interferon-induced, transmembrane and GPI-anchored protein that restricts the release of a number of enveloped viruses including all retroviruses tested as well as members of the arenavirus (Lassa) and filovirus (Ebola and Marburg) families [7]–[10]. However, how BST-2 mediates the retention of nascent virions is unclear. Viral antagonists of BST-2 include the HIV-1 Vpu, HIV-2 Env, SIV Nef, Ebola glycoprotein, and KSHV K5 proteins [4], [5], [11]–[14]. A common feature of the antagonism of BST-2 by viral gene products is its removal from the cell surface, the presumed site of virion-tethering activity.

An unusual membrane topology, localization in cholesterol enriched membrane microdomains, and homo-dimerization may each be key to BST-2's restrictive activity. BST-2 binds the lipid bilayer twice, via both an N-terminal transmembrane domain and a C-terminal GPI anchor [8]. This topology leads to the hypothesis that BST-2 retains virions by directly spanning the lipid bilayers of the virion and host cell. Many enveloped viruses including HIV-1 and Ebola bud from cholesterol-enriched membrane domains in which BST-2 is enriched [15],[16]. These observations lead to the hypothesis that BST-2 is incorporated into virions as part of the mechanism of restriction. BST-2 forms disulfide-linked dimers [6]. This observation leads to the hypothesis that the molecular topology of restriction includes dimerization between virion- and cell-associated BST-2.

Here, we show that BST-2 is positioned to directly retain virions on the surface of infected cells and is incorporated into virions. We confirm that virions retained on the cell surface can be released by proteolysis, but find that they are not released by cleavage of GPI-anchors with phosphatidyl inositol specific phospholipase C or by disulfide reduction with dithiothreitol. Although these findings leave the precise configuration of the BST-2 molecules that restrict release unsolved, they support a model in which BST-2 incorporates into virions to directly restrict their release from the plasma membrane. This mechanism may be broadly applicable to the inhibition of enveloped viruses by BST-2.

## Results

### BST-2 is present along the plasma membrane in a punctate distribution and is positioned to directly tether budding virions

**Figure 1:**
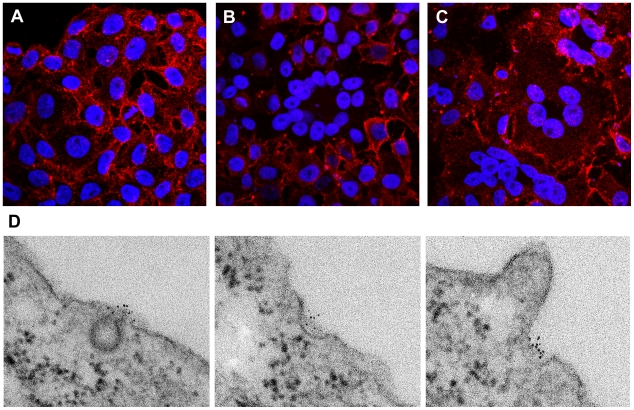
Punctate cell surface distribution of BST-2 and surface down-regulation by HIV-1 encoding the Vpu protein. HeLa cells were left untransfected (A) or transfected with a plasmid expressing the complete wild type HIV-1 genome (B) or with a plasmid expressing an HIV-1 genome lacking *vpu* (C). The next day, the cells were fixed and stained without permeabilization using an antibody to the BST-2 ectodomain and a secondary labeling system including streptavidin-conjugated cadmium selenide/zinc sulfide nanocrystals (quantum dots; Qdot 625 nm). Nuclei were stained with DAPI. Data were acquired as a Z-series of images using an Olympus laser scanning confocal microscope. The images shown are projections of the entire Z-series for each field. (D) HeLa cells (untransfected) were plated on cover glasses, then fixed in formaldehyde and stained for surface BST-2 exactly as described above using Qdot 625 nanocrystals. After staining, the cells were further fixed in glutaraldehyde and osmium tetroxide, followed by conventional embedding and processing for thin-section transmission electron microscopy. Images were obtained using a JEOL 1200C microscope operated at 80 keV.

To test the hypothesis that BST-2 is positioned along the plasma membrane appropriately to directly tether virions, we visualized the location of the molecule using correlative fluorescence and electron microscopy. To accomplish this, we labeled the surface of HeLa cells, which express BST-2 constitutively [5], with a specific antibody that recognizes the BST-2 ectodomain [17]. This antibody was secondarily labeled with cadmium selenide/zinc sulfide nanocrystals (QDots) that are both fluorescent and electron dense; this property allowed cells labeled identically to be observed by either light or electron microscopy [18]. The surfaces of cells labeled for BST-2 were characterized by a punctate staining pattern when visualized by fluorescence microscopy (Figure 1A and Figures S1 and S2). This pattern has been noted previously using routine fluorophores [9],[19]. In cells expressing HIV-1, these puncta appear to contain Gag as well as BST-2 and have been hypothesized to reflect sites of virion-formation; in uninfected cells their identity is unclear. Here, in cultures transfected to express the complete HIV-1 genome including the BST-2 antagonist gene *vpu*, some cells were characterized by reduced or absent surface staining (Figures 1B and S1). In particular, multinucleated cells resulting from virally induced cell-cell fusion were strikingly low in surface BST-2 (Figures 1B and S1), consistent with the previously described reduction in the expression of cell-surface BST-2 induced by Vpu [5]. In contrast, no reduction in surface stain was seen when cells were transfected to express a *vpu*-negative HIV-1 genome; in this case, multinucleated cells resulting from virally induced cell-cell fusion expressed abundant BST-2 on their surfaces (Figures 1C and S1). Together, these data indicated that the QDot-based stain for BST-2 revealed the previously noted punctate surface pattern, and it faithfully revealed the removal of BST-2 from the cell surface by Vpu as expressed in the context of the complete viral genome.

**Figure 2:**
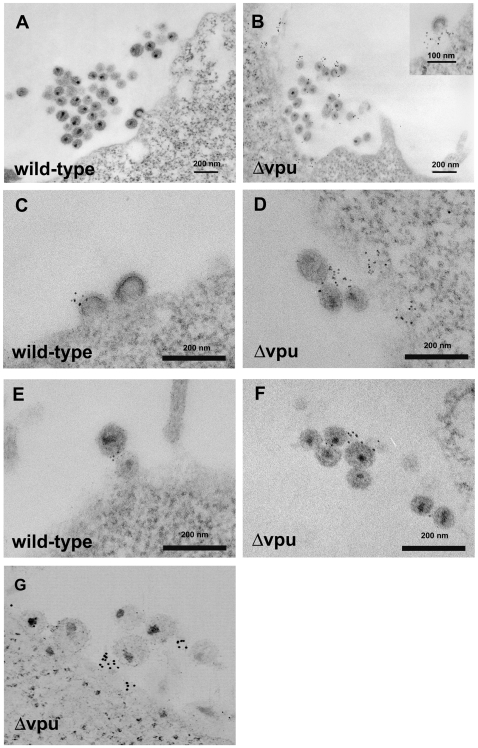
Electron microscopic evidence of direct virion tethering and virion-incorporation of BST-2. HeLa cells were transfected to express the complete HIV-1 genome (wild type; A, C, and E) or a genome lacking *vpu* (Δvpu; B, D, F, and G), then stained for cell surface BST-2 and processed for electron microscopy as described in the legend of Figure 1. Panels A-F are images of thin sections obtained using a JEOL 1200C microscope operated at 80 keV. Panel G is a projection view of a thick section (approximately 250 nm) derived from a tomographic tilt series of images obtained using an FEI Titan electron microscope operated at 300 keV.

To determine the distribution of BST-2 at the ultrastructural level and in relation to nascent HIV virions, cells stained in an identical manner to those shown in Figure 1A-C were processed for transmission electron microscopy. In uninfected cells, BST-2 was found in foci along the plasma membrane (Figures 1D and S2), which likely correspond to the puncta seen using immunofluorescence. Some of these foci were associated with endocytic pits, which appeared either coated or uncoated, whereas other foci were not associated with any apparent structure. In cells expressing the complete HIV-1 genome including *vpu*, surface labeling was often relatively sparse, even in areas of clustered viral particles (Figure 2A). Such paucity of label is consistent with the reduced surface expression visualized by fluorescence microscopy. These wild type viral particles showed both immature (crescentic electron density along the perimeter of the budding virion), as well as mature morphology (electron dense cores with occasional conical shape). In contrast, in cells expressing *vpu*-negative virus, BST-2 was readily detectable at the cell surface (Figure 2B). Furthermore, label was intercalated between the plasma membrane and nascent virions as well as between nascent virions found in clusters, most of which had a mature morphology. Occasionally, striking examples of label concentrated at the neck of budding virions in the case of *vpu*-negative virus were observed (Figure 2B, inset). These electron microscopic data indicated that BST-2 is positioned appropriately to function as a direct tethering factor.

### BST-2 is incorporated into infectious HIV-1 viral particles

To determine whether BST-2 is incorporated into virions, we looked for profiles of budding virions and for profiles of virions distant from the cell surface. Surprisingly, wild type virions were not infrequently labeled for BST-2 (Figure 2C and E; see Figure S3 for control stain). This result is consistent with functional data indicating that Vpu is not a fully effective antagonist of BST-2 [5], and it is consistent with the virion-capture and immunoblot experiments described below. In rare cases, label for BST-2 was found directly between virions that appeared linked to each other (Figure 2E). In the case of *vpu*-negative virus, label was particularly evident among and between virions caught at a distance from the plasma membrane (Figure 2D and F). Potential association of such label with the plasma membrane was excluded by electron tomography of thick sections; reconstructed three-dimensional images confirmed the presence of labeled virions that were unambiguously discontinuous with the plasma membrane (Figure 2G and Video S1). Although substantial variability was observed in the density of label for BST-2 on and between individual virions, visual inspection of 38 images yielded 358 virions with 149 virion-associated Qdots in the case of wild type virus and 327 virions with 302 associated Qdots in the case of *vpu*-negative virus, indicating a 2.3-fold greater association of label with virions in the absence of *vpu*. These immuno-electron microscopic data indicated that BST-2 is incorporated into virions. The data were also consistent with a model of viral antagonism in which Vpu decreases the density of BST-2 at sites of virion assembly and within virions themselves.

**Figure 3:**
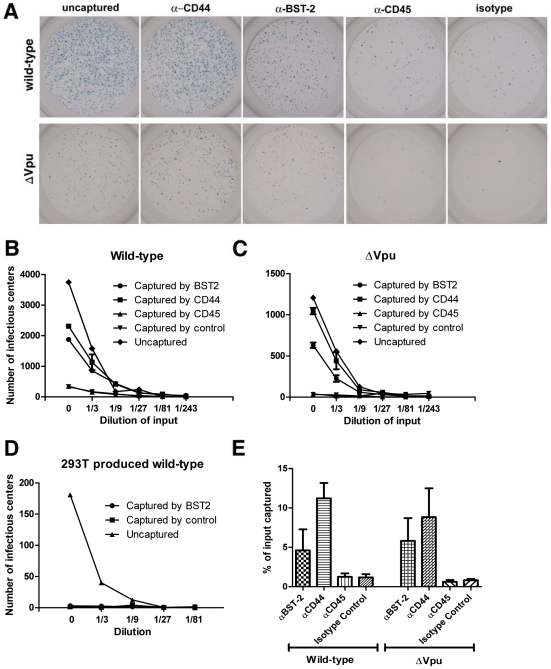
Capture of infectious HIV-1 virions using antibody to BST-2. A. HIV-1 virions were produced by transfection of HeLa cells, which express endogenous BST-2, either with a plasmid expressing the wild type HIV-1 genome or a genome lacking *vpu* (Δvpu). Forty-eight hours later, cell-free culture supernatants were collected, incubated with antibody to CD44, BST-2, CD45, or an IgG2a isotype control, then captured using magnetic microbeads coated with staphylococcal protein G. Immuno-captured virions were resuspended in the original culture volumes. Virions, uncaptured or captured using the indicated antibodies, were used to infect CD4-positive HeLa cells containing an LTR-β-galactosidase indicator construct. Two days later, infectious centers were visualized by incubation with X-gal to generate blue colored foci. Images of individual wells were acquired using a CCD camera. The wells shown are the result of infection using undiluted preparations, which were each equivalent to 200 µl of the original, uncaptured cell-free culture supernatants. The uncaptured preparation of wild type virus contained five-fold more HIV-1 p24 capsid antigen than that of Δvpu, consistent with the restriction of virion-release by endogenous BST-2 and counteraction by Vpu (data not shown). B and C. An independently prepared set of virions was produced from HeLa cells, subjected to immuno-capture, and infectivity was measured as described for panel A. Each preparation was serially diluted as shown (“0” indicates undiluted sample) and used to infect CD4-positive HeLa cells in duplicate. Infectious centers were counted using image analysis software; error bars are the actual values of duplicate wells and in some cases are too small to see. The data shown are representation of three independent experiments. D. Virions (wild type) were produced by transfection of HEK293T cells lacking endogenous BST-2, subjected to immuno-capture using antibody to BST-2 or an antibody isotype control, and infectivity was measured as in panels B and C. Data are the averages of duplicate wells; the error bars are too small to see. E. The fraction of the total viral p24 capsid antigen that was recovered in these immuno-capture experiments was measured using an ELISA. Error bars are the standard deviation of values obtained from three capture experiments using independent preparations of viruses made from HeLa cells.

To validate the incorporation of BST-2 into virions, we devised a bead-based virion-capture assay using the same antibody as was used above for the morphologic studies. A key feature of this assay is the virologic readout of infectivity, allowing confirmation that BST-2 is incorporated into bona fide infectious virions (Figure 3A-C). Preparations of cell-free virions produced from BST-2-positive HeLa cells were mixed with antibody to the BST-2 ectodomain, or with an isotype-matched control antibody, an antibody to CD44, or an antibody to CD45. The virion-antibody complexes were then captured on coated magnetic microbeads and used to infect adherent CD4-positive HeLa indicator cells in an infectious center assay of HIV-1 infectivity (Figure 3A-C). CD44 is incorporated into virions and served as a positive control for the capture [20]. CD45 is excluded from virions produced from hematopoietic cells [21], but here it serves only as a second specificity control, since CD45 is not known to be expressed on HeLa cells. Strikingly, antibody to BST-2 captured infectious virus from solution, both in the case of wild type and *vpu*-negative genomes. In contrast, infectious virus (wild type) produced from HEK293T cells, which do not express BST-2 constitutively [4],[5], was not captured by antibody to BST-2 (Figure 3D). Capture of virions produced from HeLa cells by antibody to BST-2 was confirmed by measurement of viral capsid (p24) antigen by ELISA (Figure 3E). The efficiency of capture as measured by infectivity or p24 ELISA was not significantly affected by Vpu; this suggests either that Vpu does not significantly decrease the incorporation of BST-2 into virions or that both wild type and *vpu*-negative virions incorporate a threshold amount of BST-2 sufficient for capture. Immuno-capture of three independent sets of wild type and *vpu*-negative virus preparations confirmed the incorporation of BST-2 into infectious virions of HIV-1 (Figure 3 and data not shown).

**Figure 4:**
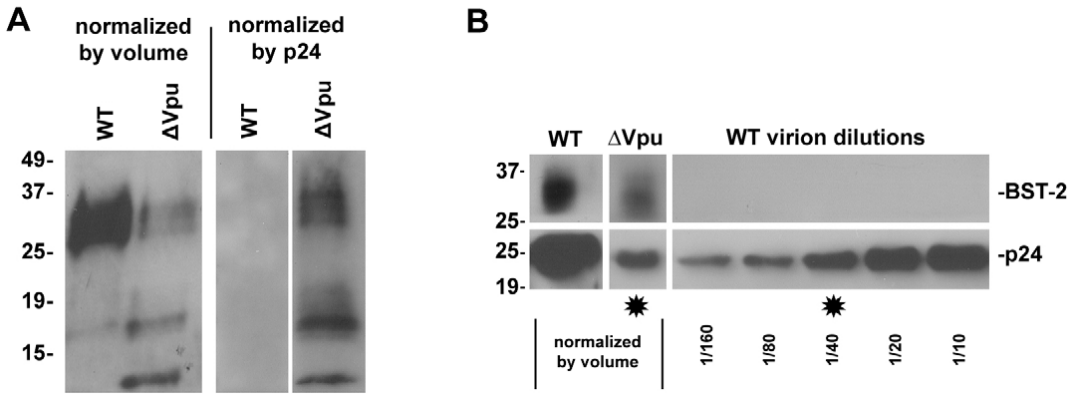
Virion-associated BST-2 detected by immunoblot. HIV-1 virions were produced by transfection of HeLa cells, which express endogenous BST-2, either with a plasmid expressing the wild type HIV-1 genome or a genome lacking *vpu*. Twenty-four hours after transfection, culture supernatants were centrifuged at 800 *g* for 8 minutes to remove cellular debris. Virions were concentrated by centrifugation of the clarified supernatants at 23,000 *g* for three hours, and then resuspended in SDS- and DTT-containing buffer for quantitation of p24 capsid by ELISA and for analysis of BST-2 and p24 content by western blot. A. Blots were probed for BST-2. Left lanes were normalized by volume: the wild type sample contained 85 ng of p24 antigen, whereas the Δvpu sample contained 4 ng of p24 antigen. The greater p24 content of the wild type preparation reflects the antagonism of BST-2 mediated restriction of virion-release by Vpu. Right lanes were normalized by p24 antigen content (5 ng for each sample as determined by ELISA). B. Samples analyzed in A were reanalyzed including a dilution series of wild type virions and detection of p24 antigen by immunoblot. Stars indicate lanes with approximately equal content of p24. Western blots for BST-2 were performed as previously described [19]. Molecular weights are in kilodaltons.

**Figure 5:**
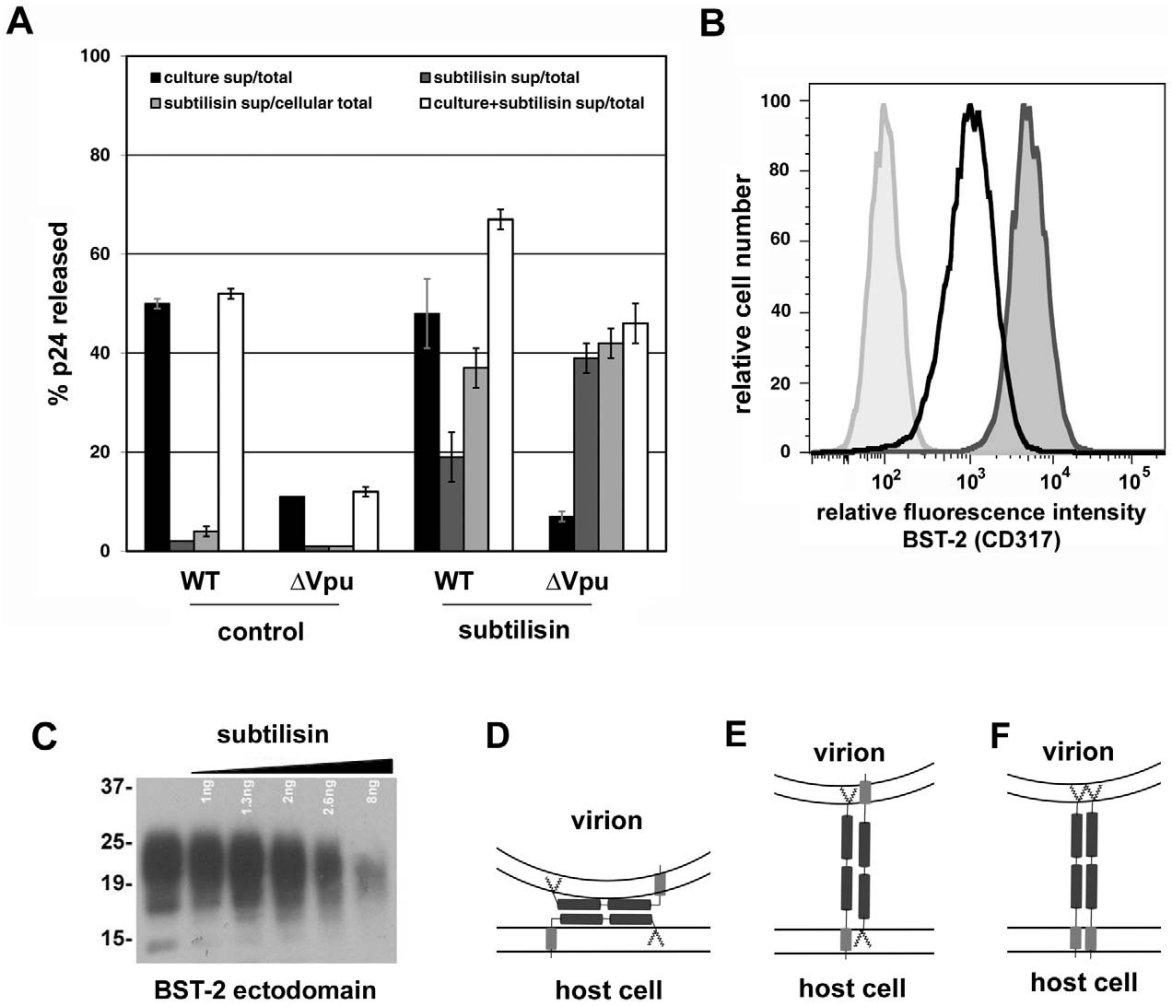
Release of nascent virions and the BST-2 ectodomain by proteolysis of the cell surface with subtilisin; models of direct tethering. A. HeLa cells were transfected to express the complete HIV-1 genome (WT) or a genome lacking *vpu* (ΔVpu). The next day, culture supernatants were removed to measure the amount of spontaneously released virions (“culture sup”). The cells were then incubated with buffer (control) or buffer containing 1 mg/ml subtilisin for 15 minutes at 37C. Proteolysis was stopped by the addition of PMSF to 5 mM; the cells were pelleted, and the supernatants were removed to measure the amount of virions released (“subtilisin sup”). The cells were then directly lysed into Triton-X 100 containing buffer to measure cell-associated viral antigen. The concentrations of HIV-1 p24 capsid antigen were measured in all samples by ELISA. “Total” indicates the total p24 antigen produced by the culture, i.e., the sum of that released spontaneously, after incubation with buffer or subtilisin, and cell-associated. “Cellular total” indicates the amount of p24 antigen initially associated with the cells, i.e., the sum of that released by buffer or subtilisin and cell-associated. “Culture+subtilisin sup” indicates the total amount of p24 antigen releasable from the cell surface, i.e., the sum of that released spontaneously and after incubation with buffer or subtilisin. Data are the percentages of p24 released; error bars indicate the values of duplicate measurements. B. HeLa cells were treated with buffer or subtilisin as above; proteolysis was quenched as above; and the cells were stained with antibody to the BST-2 ectodomain or with an isotype control antibody, and then analyzed by flow cytometry. Lightly shaded curve, antibody isotype control; unshaded curve, BST-2 after proteolysis of the cell surface using subtilisin; darkly shaded curve, BST-2 on untreated cells. C. Soluble BST-2 ectodomain (residues 49–180 as described previously [6]) was incubated with the indicated amounts of subtilisin for fifteen minutes before analysis by SDS-PAGE and detection by immunoblot with antibody specific to the BST-2 ectodomain. D. Model of ectodomain interaction. E. Model of virion-cell membrane spanning dimer: anti-parallel orientation. F. Model of virion-cell membrane spanning dimer: parallel orientation.

**Figure 6:**
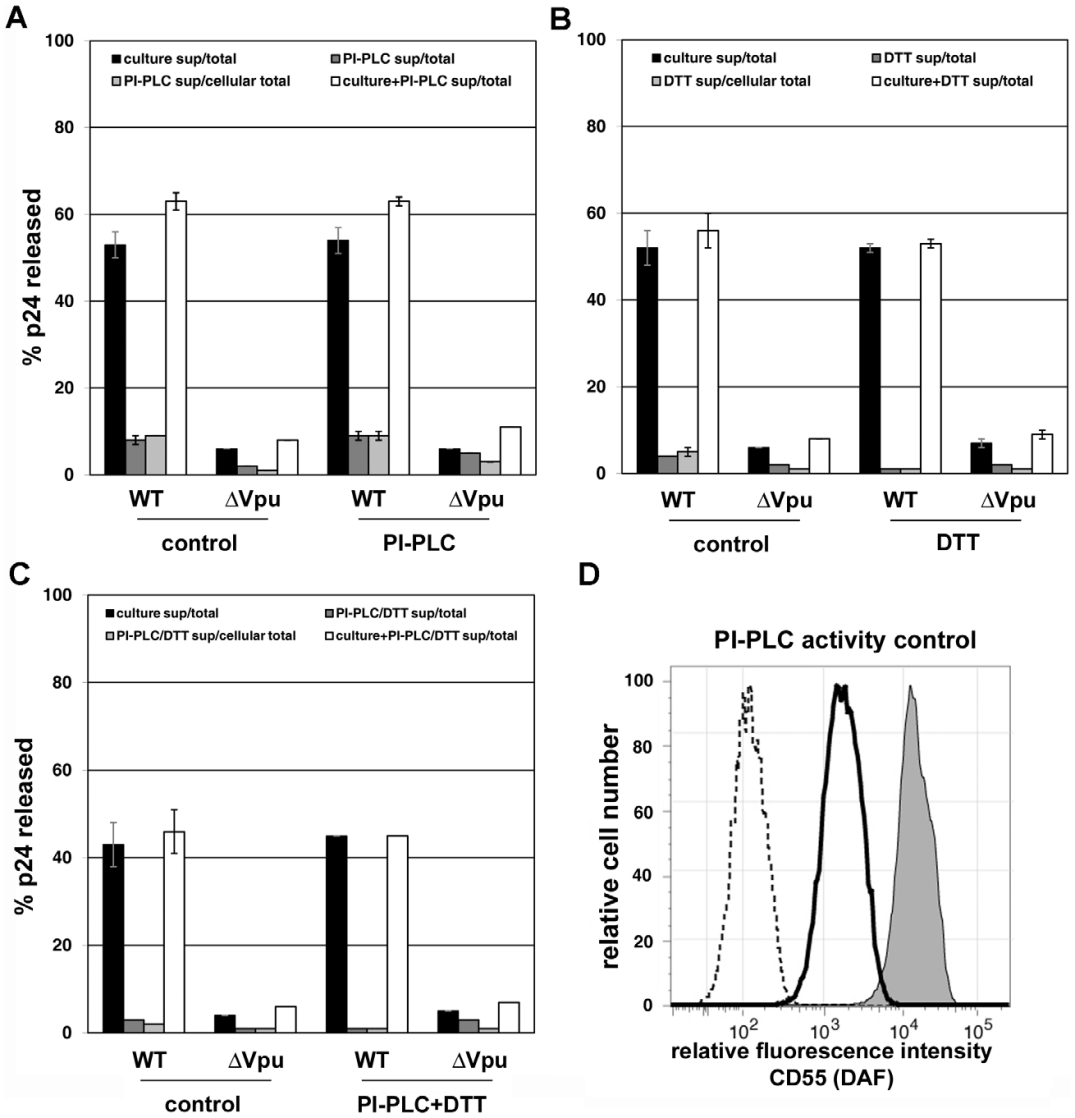
Persistence of virion tethering despite cleavage with PI-specific PLC either with or without disulfide reduction. Experiments were performed as described in the legend of Figure 5, except that rather than subtilisin, specific treatments were used to test models of tethering. A. Release data using bacterial phosphatidyl inositol-specific phosphoplipase C (PI-PLC; 2.5 U/ml). B. Release data using 5 mM dithiothreitol (DTT) at 37C. C. Release data using PI-PLC followed by DTT. In panels A-C, data are the percentages of p24 released; error bars indicate the values of duplicate measurements. D. Confirmation of PI-PLC activity using CD55 (decay accelerating factor) as a control protein. HeLa cells were stained with either a FITC-conjugated isotype control antibody (dotted line), or a FITC conjugated specific antibody to CD55 (dark-outlined open curve and shaded curve), before analysis by flow cytometry. Cells treated with PI-PLC (2.5 U/ml) are indicated by the dark-outlined open curve, whereas control cells incubated in buffer only are indicated by the shaded curve.

Immunoblot analysis also supported the conclusion that BST-2 is incorporated into virions and further suggested that Vpu inhibits this (Figure 4, in which virions produced from HeLa cells and concentrated by centrifugation were analyzed). Remarkably, when normalized by the volume of the original culture supernates (Figure 4A, left panel), preparations of wild type virions contained more BST-2, as well as more p24 capsid protein, than virions produced by *vpu*-negative virus. This difference in BST-2 contents in the volume-normalized samples suggests that the signal was derived from virions and not merely cellular debris or exosomes; if the latter were the case, then the volume normalized samples from cultures expressing wild type virus should have contained less BST-2, due to Vpu-mediated down-regulation. In contrast, when the preparations of virions were normalized by their contents of p24 antigen, BST-2 was essentially only detectable in the absence of Vpu (Figure 4A and B). The apparent association of BST-2 with virions and a relative decrease in the content of BST-2 in virions produced in the presence of Vpu was observed in three independent preparations. These observations were robust to filtration of the virion preparations through 0.22 µM pore size membranes, suggesting that the detection of BST-2 was not due to the presence of aggregates of BST-2-containing cellular vesicles and virions (data not shown). Interestingly, the relatively greater phenotype of *vpu* detected in this assay (an apparent 40-fold increase in virion output) as compared to that detected by measurement of p24 in unfractionated culture supernatants by ELISA (a 5–8-fold increase; see Figures 5 and 6) may be due to a reduced fraction of pelletable p24 when virions are produced in the absence of Vpu (data not shown). Notably, virions produced in the absence of Vpu contained, in addition to a triplet of species that migrated with apparent molecular mass in the range of 27–37 kDa (likely representing heterogeneously glycosylated BST-2), two bands of under 20 kDa in apparent mass. These species are less than the predicted size of unmodified BST-2 (20 kDa), and their identity is unknown; conceivably, they could represent proteolysis of higher mass forms. Overall, these immunoblot data, like the results of immuno-electron microscopy and immuno-capture, support the conclusion that BST-2 is incorporated into virions. Furthermore, the immunoblot results suggest that Vpu reduces the virion-incorporation of BST-2.

### Release of nascent virions from the surface of infected cells by proteolysis of the BST-2 ectodomain supports a direct tethering model of restriction

To support further a direct tethering model, we confirmed that proteolysis with subtilisin releases virions retained on the cell surface [Figure 5A, in which the black bars indicate the fraction of the total amount of p24 capsid antigen produced by the culture that was spontaneously released into the medium after transfection with wild type or *vpu*-negative (Δvpu) proviral plasmids; the dark gray bars indicate the fraction of the total that was further eluted from the cells with buffer (control) or subtilisin; and the light gray bars indicate the fraction of the cell-associated p24 that was eluted with buffer or subtilisin] [22]. The fractional elution of p24 was greater in the absence of Vpu, consistent with a greater number of virions initially retained at the cell surface. Notably, these quantitative data indicated that the total fraction of p24 releasable from the cells (adding that released spontaneously to that released by proteolysis with subtilisin; open bars in Figure 5A) is greater in the case of wild type than Δvpu. The “non-releasable” p24 in the case of cells expressing *vpu*-negative virus presumably reflects virions that have been endocytosed subsequent to restricted release and are not accessible to proteolysis. We further showed that proteolysis with subtilisin indeed acts on BST-2; it largely removed the BST-2 ectodomain from the cell surface as detected by flow cytometry (Figure 5B), and it degraded the ectodomain in vitro (Figure 5C). These results are consistent with direct tethering mediated by BST-2, but they do not discriminate among several potential topological models of restriction (Figure 5, D-F).

### The topology of virion restriction: neither PI-PLC nor DTT releases nascent virions from the surface of infected cells

The preceding data suggest that the incorporation of BST-2 into viral envelopes and a direct tethering mechanism underlie its restrictive activity. However, the topology of the BST-2 molecules that mediate the retention of nascent virions remained unclear. One hypothesis is that virion-associated BST-2 interacts directly with cell surface BST-2, potentially via disulfide bonds but alternatively via predicted coiled-coil regions in the ectodomain of the protein (Figure 5D and [4]). Alternatively, one end of BST-2 could embed in the lipid bilayer of the cell and the other in that of the virion. Such membrane-spanning models are depicted in Figure 5E and F; notably, BST-2 dimers could span the virion and host membranes in parallel or anti-parallel orientations.

Here, release of nascent virions was not obtained by incubation of virus-producing cells with phosphatidyl inositol (PI)-specific phospholipase C (PLC), which is expected to cleave the GPI-anchor of BST-2 (Figure 6A). Because BST-2 remains attached to the cell surface by its transmembrane domain after cleavage of its GPI anchor (data not shown), PI-PLC activity was validated using CD55 (decay accelerating factor), which is a typical GPI-anchored protein (Figure 6D; in which PI-PLC effectively removed CD55 from the cell surface). These data weighed against the membrane-spanning parallel dimer model of Figure 5F. Incubation of cells with dithiothreitol (DTT) to reduce disulfide bonds also failed to release virions (Figure 6B), weighing against a self-interaction mechanism mediated exclusively by disulfide bonds. Incubation with PI-PLC followed by DTT (Figure 6C) also failed to release virions, weighing against an anti-parallel, disulfide linked, membrane-spanning model (Figure 5E). These data do not provide direct support for any specific topology of restriction, but they leave open the possibility that the model shown in Figure 5D is operative via a coiled-coil based interaction between the ectodomains of virion- and cell-associated BST-2.

## Discussion

The interferon induced, GPI-anchored and transmembrane protein BST-2 restricts the release of enveloped virions from infected cells by an unclear mechanism. Here, the prototypic restricted virus, HIV-1, and the prototypic viral antagonist protein, Vpu, were used to investigate this mechanism. The data provide key initial support for a model in which BST-2 is a direct tethering factor that is itself incorporated into infectious virions.

Recent reports have questioned the incorporation of BST-2 into virions and the co-localization of BST-2 with virion proteins, leaving a direct tethering model of restriction unsupported [23],[24]. An inability to detect BST-2 in virions by immunoblot may be attributable to insufficient sensitivity of the assay, whereas it is more difficult to explain the reported negative data for co-localization. Here, a combination of morphologic, virologic, and biochemical approaches provided evidence supporting direct tethering and virion-incorporation of BST-2.

Evidence that BST-2 is incorporated into virions was provided by immuno-electron microscopy, immuno-capture of infectious virions, and routine immunoblot. The immuno-electron microscopic data specifically localized BST-2 as adjacent to virions, between virions and the plasma membrane, and in rare instances between virions that were linked to each other. The electron microscopic data also suggested that the punctate distribution of BST-2 seen at the cell surface by fluorescence microscopy is only partly due to the occurrence of the protein in endocytic pits. Many of the foci seen along the plasma membrane were not associated with any apparent structure. Intriguingly, these foci could represent membrane microdomains containing BST-2, such as cholesterol-enriched lipid rafts, although we cannot exclude that they reflect antibody-induced lateral aggregation of BST-2 within the lipid bilayer.

Somewhat surprisingly, immuno-electron microscopy, immuno-capture of infectious virions, and routine immunoblot each indicated that virions produced in the presence of Vpu are not devoid of BST-2. However, immunoblot, and to a lesser extent electron microscopy, suggested that Vpu decreases the amount of BST-2 in virions. Notably, antibody to the BST-2 ectodomain captured virions produced in the presence or absence of Vpu equally well; this may reflect a threshold amount of virion-associated BST-2 required for immuno-capture that is met by virions produced in either context. Altogether, these data indicate the presence of BST-2 in virions. The data also support a relative but not absolute exclusion of BST-2 from virions by Vpu.

One of two topological models has seemed likely to explain restriction mediated directly by BST-2: a membrane spanning model in which BST-2 embeds one end in the cell membrane and the other in the virion membrane, or a self-interaction model in which virion-associated and cell-surface-associated BST-2 molecules interact via their ectodomains. Here, we found no direct support for membrane spanning models; phosphatidyl inositol-specific phospholipase C (PI-PLC), either with or without disulfide reduction, failed to release virions retained on the surface of BST-2-expressing cells. These results weigh against membrane spanning models involving parallel dimers or anti-parallel dimers held together by disulfide bonds. A caveat to this interpretation is that the failure of PI-PLC to release virions could be due to modification of the GPI anchor of BST-2 such that it is resistant to this enzyme, a possibility not excluded by the enzyme activity control herein (release of CD55). As noted above, PI-PLC did not release BST-2 from the cell surface, consistent with membrane anchoring by the protein's transmembrane domain (data not shown); consequently, we could not validate the activity of PI-PLC on native BST-2. Similarly, the failure of reduction with DTT to release virions could be due to an inability of DTT to reach the key disulfide bond(s) at physiological temperature, for example, if they are protected within a well-folded structure. Similar attempts to release virus-like-particles of Ebola retained on the cell surface by BST-2 with as much as 500 mM DTT were also ineffective [14]. Recent mutational analyses of cysteine residues within the BST-2 ectodomain suggest that disulfide-mediated dimerization is a correlate of the restriction of HIV-1 release, but not of arenavirus release [25],[26]. Notwithstanding these potentially conflicting findings, exactly how disulfide mediated dimerization would contribute to restriction, if not by mediating an interaction between virion- and cell-associated BST-2, is unclear.

A direct restriction mechanism that is not disfavored by any of the data herein is an ectodomain self-interaction model such as that shown in Figure 5D, but in which the driving force of tethering is a coiled-coil based interaction. To test this hypothesis, the role of the predicted coiled-coil region in the ectodomain needs to be directly and critically evaluated: are key residues in the predicted structure required for restriction? Does the putative interaction responsible for restriction involve two, three, four, or more α-helices?

Although each of the models of Figure 5 may be too simplistic, a self-interaction model of restriction is attractive: a single plasma membrane protein, BST-2, is localized to sites of viral assembly, incorporates into virions, and dimerizes or forms higher order multimers or aggregates to restrict release. This direct tethering, self-interaction model of restriction relies only on the localization of BST-2 to sites of viral budding and on the incorporation of BST-2 into virions. Consequently, it can potentially be generalized to all enveloped viruses that assemble on membrane microdomains that contain BST-2. Conversely, a model of relief of restriction by removal of BST-2 from the sites of viral assembly and from virions themselves can potentially be generalized to all viral proteins that decrease the expression of BST-2 at the plasma membrane. So far, such proteins include HIV-1 Vpu, HIV-2 Env, SIV Nef, and KSHV K5 [5], [11]–[13].

Notably, the data herein indicate the presence of BST-2 in infectious HIV-1 virions that are spontaneously released from cells, even when the viral antagonist protein Vpu is expressed. The HeLa cells used for the studies herein express BST-2 endogenously, obviating transient expression methods that may be prone to artifactual over-expression of BST-2 in individual cells. On the other hand, whether wild-type virions produced from primary T cells or produced in vivo contain BST-2 remains to be determined. The observations herein suggest the possibility that virion-associated BST-2 serves functions in addition to tethering nascent virions. In this regard, we note the potential for virion-associated BST-2 to interact with ligands, including itself, on immune effector cells. BST-2 is constitutively expressed, at least in mice, on plasmacytoid dendritic cells (PDCs), as well as on B cells and activated T cells in humans [7],[27]. Considering the incorporation of BST-2 into virions and the potential for interaction between virion- and cell-associated BST-2, we speculate that in addition to its direct antiviral activity as a tetherin, BST-2 might flag enveloped viruses for subsequent binding to PDCs and B-cells, which are antigen-presenting cells, and so stimulate the host adaptive immune response. Recently, BST-2 was identified as a ligand for a receptor on PDCs, ILT7, which transduces a signal for shut-off of interferon production [28]. Based in these findings and the data herein, we also speculate that virion-associated BST-2 might provide negative feedback to the interferon response. These mechanisms would place BST-2 at the interface of innate and adaptive immunity to enveloped viruses.

In conclusion, the data herein advance a direct model of restriction in which BST-2 is found both at the sites of viral assembly along the plasma membrane and within budding and nascent virions. While this paper was being finalized, independent evidence for direct restriction of virus release and virion-incorporation of BST-2 was reported [29]. Biochemical data suggested a parallel dimer configuration. Strikingly, an “artificial tetherin” that lacks primary sequence homology with BST-2, but which contains its key membrane binding and structural features, showed antiviral activity, indicating that no cellular cofactors are likely obligatory for the tethering phenomenon [29]. Directly relevant to the data herein, a mutant BST-2 lacking a GPI-anchor site was incorporated into virions but was unable to restrict virion release. These observations would leave open the role of the GPI-anchor in restriction, if not as one of the two membrane anchors in virion-cell membrane spanning models. On the other hand, this study directly demonstrated a requirement for the coiled-coil ectodomain of BST-2 in restriction and showed that a heterologous, dimeric coiled-coil could provide restrictive activity. These data can be interpreted to support a coiled-coil based self-interaction model of restriction. Alternatively, as proposed by the authors, the coiled-coil structure could be needed for an extended conformation of the ectodomain, which might facilitate spanning of the virion- and cell-membranes [29]. While the molecular topology of the BST-2 molecules that restrict virion release thus remains to be resolved, the augmentation of BST-2 activity and the inhibition of viral antagonists such as Vpu likely represent new approaches to the prevention and treatment of infections due to enveloped viruses. The development of these approaches depends on understanding the regulation of BST-2 during the immune response as well as on deciphering the structural basis of virion tethering and of the action of viral proteins that antagonize BST-2 function.

## Materials and Methods

### Plasmids, antibodies, and reagents

The proviral plasmid pNL4–3 was obtained from the National Institutes of Health (NIH) AIDS Research & Reference Reagent Program and contributed by Malcolm Martin [30]. The pNL4-3 mutant ΔVpu (vpuDEL-1) was provided by Klaus Strebel [31]. The murine monoclonal antibody to BST-2/HM1.24/CD317 and the BST-2 ectodomain protein were gifts from Chugai Pharmaceutical Co., Kanagawa, Japan [17]. For flow cytometry, an IgG2a antibody isotype control, a goat, anti-mouse IgG antibody conjugated to allophycocyanin (APC) and a FITC-conjugated antibody to CD55/DAF were obtained from BioLegend (San Diego, CA). For immunofluorescence and immuno-electron microscopy, a goat anti-mouse IgG antibody conjugated to biotin was obtained from Jackson ImmunoResearch (West Grove, PA), and streptavidin-conjugated cadmium selenide/zinc sulfide nanocrystals (quantum dots; QDot 625) were obtained from Invitrogen (Carlsbad, CA). Subtilisin was from Sigma-Aldrich. PI-specific phospholipase-C was from Prozyme, San Leandro, CA or Sigma-Aldrich.

### Cells and transfections

The HeLa cells used in this study were clone P4.R5, which express both CD4 and CCR5 and were obtained from Ned Landau; these cells are a derivative of clone P4 and were maintained in DMEM plus 10% fetal bovine serum (FBS), penicillin/streptomycin, and puromycin [32]. HEK293T cells were also obtained from Ned Landau and were maintained in EMEM plus 10% FBS and L-glutamine. Cells were transfected using Lipofectamine2000 (Invitrogen) according to the manufacturer's instructions. For the microscopic experiments, cells were transfected in MatTek glass bottom dishes, using 0.8 µg pNL4-3 or pvpuDel-1. For production of virions, cells were plated in 10 cm tissue culture dishes and transfected with 16 µg of pNL4-3 or pvpuDel-1.

### Electron and fluorescence microscopy

HeLa P4.R5 cells were plated on coated MatTek glass bottom dishes and transfected as indicated above. One day after transfection, the cells were fixed using 3% formaldehyde in PBS and stained using the murine monoclonal antibody to BST-2/HM1.24/CD317 (0.05 µg/ml), followed by goat anti-mouse-biotin (1∶100) and streptavidin-QDot 625 (1∶100). For fluorescence microscopy, cells were mounted in anti-fade media containing DAPI, and image data were obtained using an Olympus laser scanning confocal microscope. Z-series of two-channel images were colored, merged and projected using Image J. For transmission electron microscopy, parallel samples were re-fixed in 2% glutaraldehyde (EM Sciences) in 100 mM sodium cacodylate buffer (pH 7.4) for 30 min, post-fixed in 1% osmium tetroxide for 30 min, stained in 2% uranyl acetate in water for 1 h, dehydrated in an ethanol gradient, and embedded in Durcupan ACM (Fluka). Thin sections were stained with Sato's lead. Micrographs were obtained using a JEOL 1200 transmission electron microscope operated at 80kV. For electron tomography, sections of approximately 250 nm thickness were stained with Sato's lead and 2% uranyl acetate. Series of micrographs were collected on a FEI Titan transmission electron microscope at 300 keV while the sample was tilted from −60° to +60° in 2° increments. The micrographs were digitized and aligned using IMOD software [33]. A transform-based back projection software package was then used to create the final alignment and back projection resulting in a three-dimensional volume [34].

### Virus production

Virus was produced from either HeLa P4.R5 cells or HEK 293 T cells. Cells (6×10^6^) were plated in 10 cm tissue culture dishes and transfected as described above with 16 µg of either pNL4-3 or pvpuDel-1. Virus-containing culture supernatants were harvested 48 hours later and clarified by centrifugation at 400×*g* to remove cellular debris.

### Virus immuno-capture

Clarified culture supernatants were incubated with antibodies as indicated and then complexed to protein G-coated magnetic microbeads (Miltenyi Biotec, Bergisch Gladbach, Germany) according to the manufacturer's instructions. Bead-bound virions were captured using Miltenyi magnetic columns, washed, and eluted using DMEM plus 10%FBS and penicillin/streptomycin/puromycin in the same volume as the input supernatant. Viral protein levels in the eluate were determined using p24 capsid ELISA (Perkin-Elmer).

### Infectivity assay

Infectious center assays of viral infectivity were performed using HeLaP4.R5 indicator cells as targets. Infected foci were developed with X-gal, imaged using a CCD camera, and quantified using image analysis software, as described previously [35].

### Flow cytometry

For analysis of surface levels of BST-2, cells were stained before fixation in phosphate buffered saline (PBS) including sodium azide and 2% FBS at 4°C using an indirect method to detect BST-2: the HM1.24 murine monoclonal antibody (0.1 µg/ml) was followed by a goat anti-mouse IgG conjugated to APC. The gate for BST-2 was set using an antibody isotype control (IgG2a) as the primary antibody. For measurement of CD55, cells were stained before fixation either with a FITC-conjugated isotype control or a FITC-conjugated anti-CD55. Cells were gated by forward and side-scatter characteristics. Composite data profiles were created using FlowJo software (Tree Star, Inc.).

### Virion-release assays

A p24 antigen capture ELISA (Perkin-Elmer) was used to determine the concentration of viral capsid protein in culture supernatants that were first clarified by centrifugation at 400 *g* as well as the concentration of capsid protein in detergent lysates (0.5% Triton-X-100 in PBS) of the adherent cells. The percentage of p24 capsid secreted into the culture media was determined as the concentration of p24 antigen in the supernatants divided by the concentration of p24 antigen in the total culture (supernatant plus cells) ×100. Additional experimental details are within the figure legends.

## Supporting Information

Figure S1 Distribution of cell surface BST-2 visualized by Qdot staining and fluorescence microscopy. HeLa cells were transfected (or not) to express wild type or *vpu*-negative (Δvpu) HIV-1, then stained without permeabilization for surface BST-2 using Qdots as described in the legend of Figure 1 and in the Materials and Methods section. A control in which a non-specific isotype-matched antibody was used instead of the antibody to the BST-2 ectodomain is shown in the left-most panel. As in Figure 1, foci of cells expressing virus are identifiable as multinucleated, syncytial cells. In the case of wild type, such foci have reduced or absent stain for surface BST-2. In contrast, the stain for BST-2 is undiminished in multinucleated cells expressing *vpu*-negative virus.
(3.20 MB TIF)

Figure S2 Distribution of BST-2 along the plasma membrane of uninfected cells. HeLa cells were stained for surface BST-2 using Qdot 625 as the label and processed both for immunofluorescence (A) and for routine transmission electron microscopy (B) as described in the legend of Figure 1. Here, the higher magnification fluorescence image more clearly shows the punctate nature of the stain, which appears as foci of clustered Qdots along the plasma membrane by electron microscopy.
(2.78 MB TIF)

Figure S3 Stain control for immuno-electron microscopy: wild-type. HeLa cells expressing the wild type viral genome were processed for immuno-electron microscopy using the Qdot -based stain as described in the legend of Figure 2 (panels A-F), except that a non-specific isotype-matched antibody was used instead of the antibody to the BST-2 ectodomain. Unlike the images of wild-type virions stained with the anti-BST-2 in Figure 2 panels C and E, the virions stained with the isotype control are virtually devoid of label.
(5.50 MB TIF)

Video S1 Tomographic reconstructed view of cell surface BST-2 in relation to budding virions. HeLa cells were transfected to express HIV-1 lacking *vpu*, then stained with antibody to BST-2 and processed for transmission electron microscopy as described in Figure 1, except that 250 nm thick sections were cut and a tilt series of images was obtained for tomography using an FEI Titan electron microscope operated at 300 keV. The initial segment of the movie is the raw tilt sequence, which begins with zero degree tilt, goes to +60 degrees, then to -60 degrees, and ends again at 0 degrees. The tilt sequence transitions into a slice-by-slice back projected volume, moving through 380 computed slices. The final segment of the movie is a maximum intensity projection (MIP) rotated about the Y-axis 720 degrees. The tilt sequence was acquired using FEI automated acquisition software. The slice-by-slice back projected volume was processed with the IMOD software package. MIP visualization and movie production was done using Visage Imaging's Amira software package.
(7.54 MB MOV)
