# Supplementary figures and images for: Direct Restriction of Virus Release and Incorporation of the Interferon-Induced Protein BST-2 into HIV-1 Particles
Source: PLoS Pathog. 2010 Mar 5;6(3):e1000701. doi: 10.1371/journal.ppat.1000701 (PMC2832767; doi:10.1371/journal.ppat.1000701)

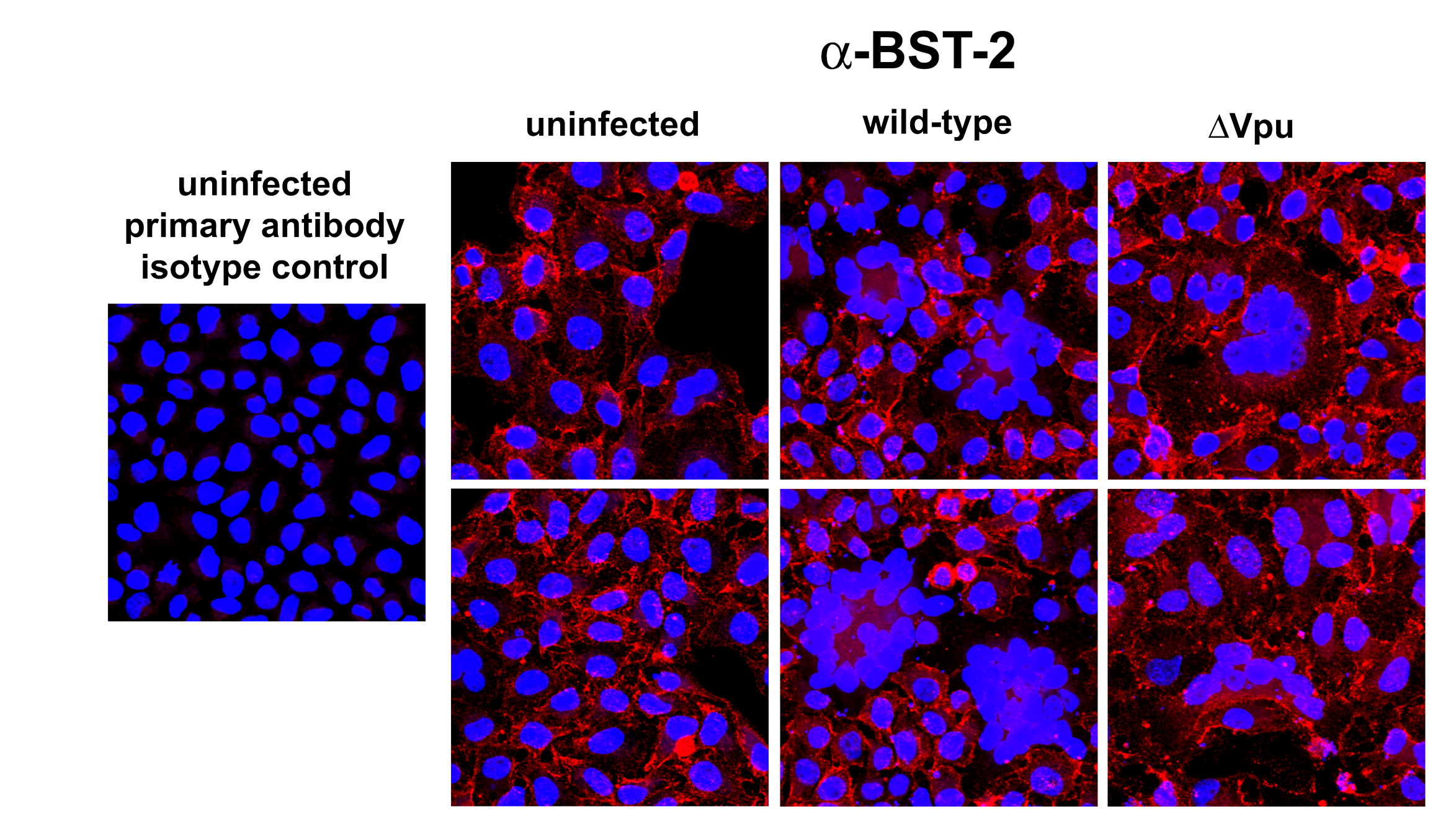

Supplement: Figure S1 — Distribution of cell surface BST-2 visualized by Qdot staining and fluorescence microscopy. HeLa cells were transfected (or not) to express wild type or vpu-negative (Δvpu) HIV-1, then stained without permeabilization for surface BST-2 using Qdots as described in the legend of Figure 1 and in the Materials and Methods section. A control in which a non-specific isotype-matched antibody was used instead of the antibody to the BST-2 ectodomain is shown in the left-most panel. As in Figure 1, foci of cells expressing virus are identifiable as multinucleated, syncytial cells. In the case of wild type, such foci have reduced or absent stain for surface BST-2. In contrast, the stain for BST-2 is undiminished in multinucleated cells expressing vpu-negative virus. (3.20 MB TIF) [file ppat.1000701.s001.tif]

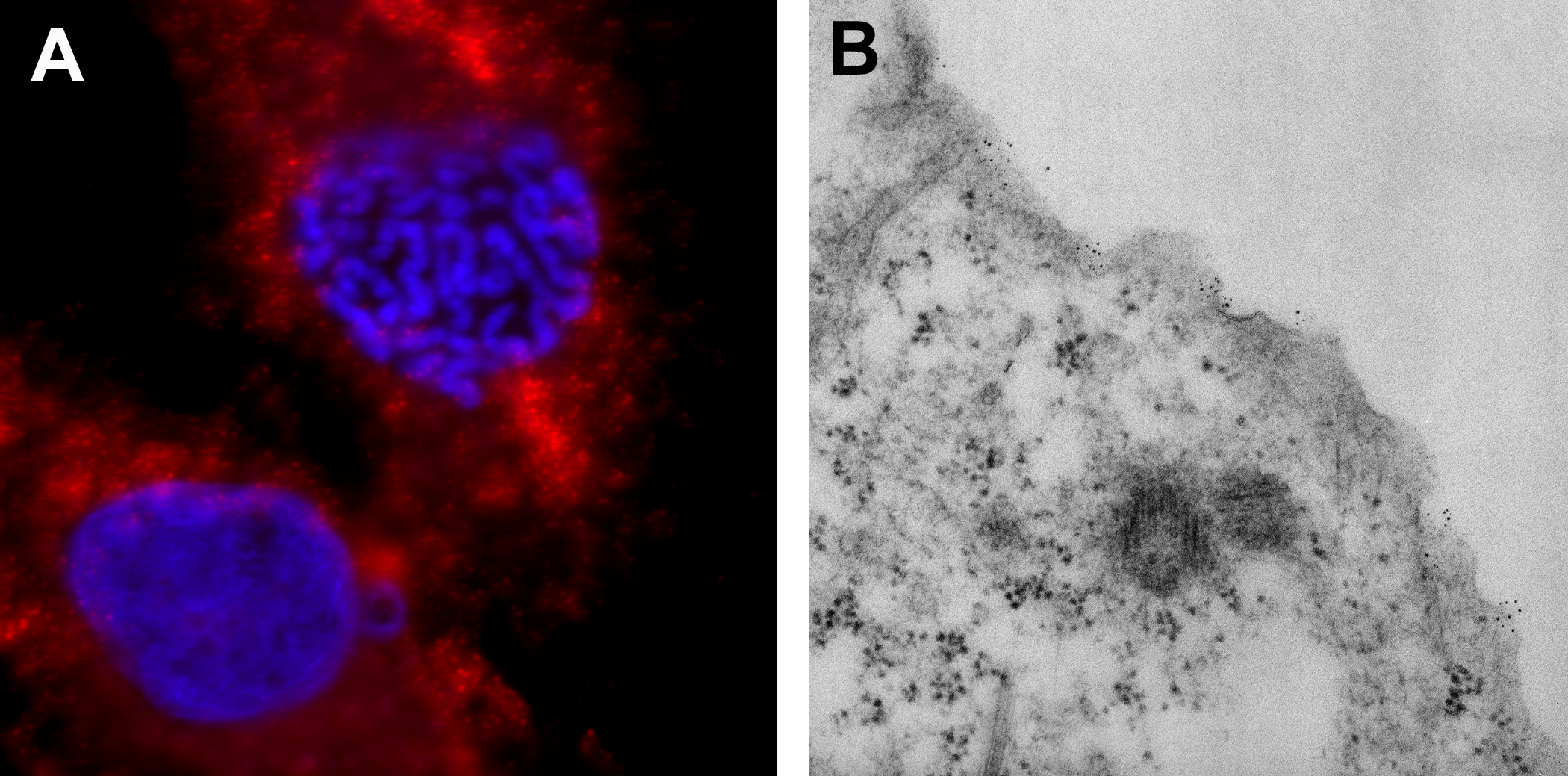

Supplement: Figure S2 — Distribution of BST-2 along the plasma membrane of uninfected cells. HeLa cells were stained for surface BST-2 using Qdot 625 as the label and processed both for immunofluorescence (A) and for routine transmission electron microscopy (B) as described in the legend of Figure 1. Here, the higher magnification fluorescence image more clearly shows the punctate nature of the stain, which appears as foci of clustered Qdots along the plasma membrane by electron microscopy. (2.78 MB TIF) [file ppat.1000701.s002.tif]

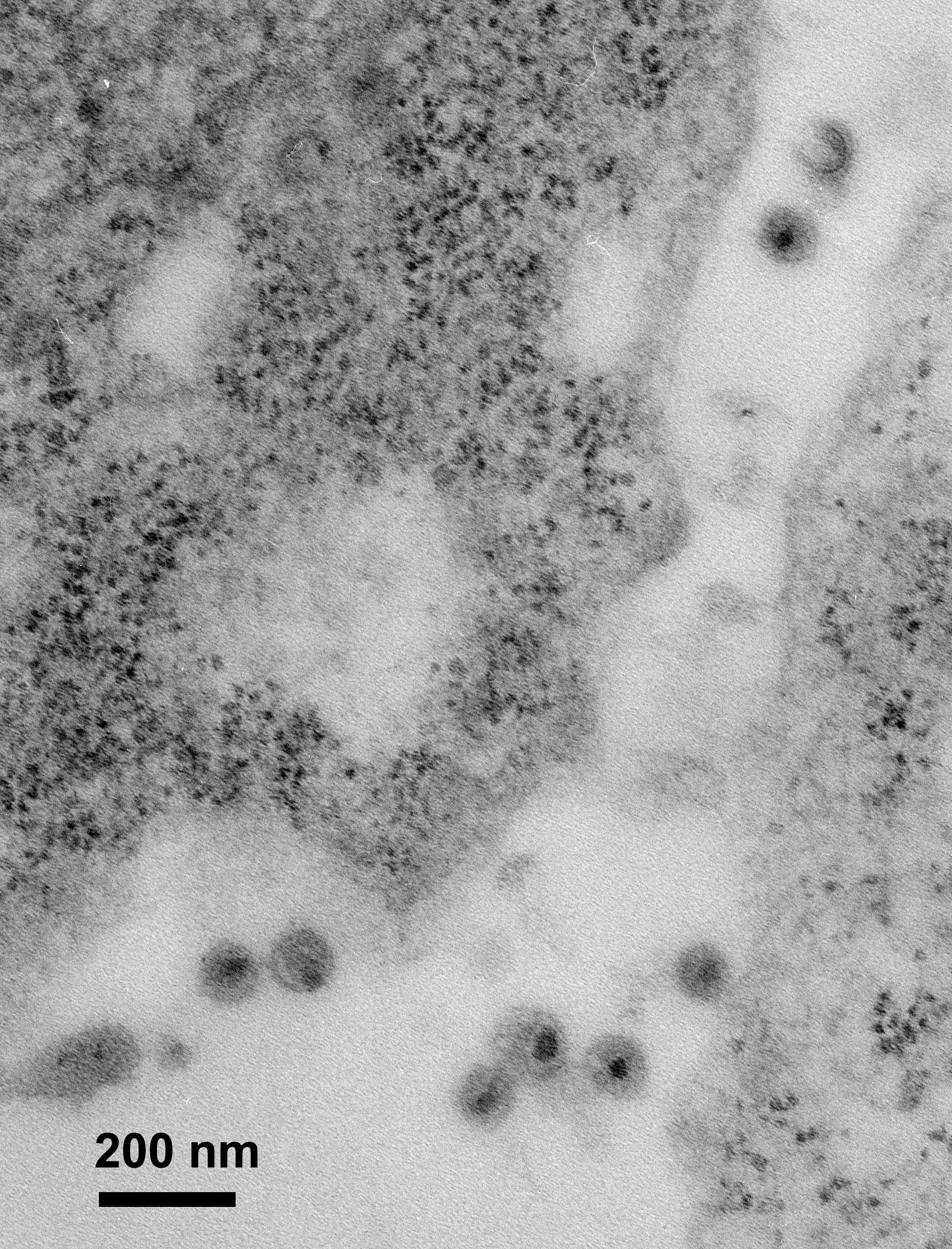

Supplement: Figure S3 — Stain control for immuno-electron microscopy: wild-type. HeLa cells expressing the wild type viral genome were processed for immuno-electron microscopy using the Qdot -based stain as described in the legend of Figure 2 (panels A-F), except that a non-specific isotype-matched antibody was used instead of the antibody to the BST-2 ectodomain. Unlike the images of wild-type virions stained with the anti-BST-2 in Figure 2 panels C and E, the virions stained with the isotype control are virtually devoid of label. (5.50 MB TIF) [file ppat.1000701.s003.tif]
